# The Consequences of Treating Asymptomatic Malaria Parasitemia

**DOI:** 10.1093/cid/ciw852

**Published:** 2017-02-08

**Authors:** Nicholas J. White

**Affiliations:** 1 Professor of Tropical Medicine, Faculty of Tropical Medicine, Mahidol University, Bangkok, Thailand

**Keywords:** *P. falciparum*, malaria risk, malaria therapy, treatment regimens.

**(See the Major Article by Portugal et al on pages 645–53.)**

Immunity to malaria is hard won, and yet it is imperfect. In areas of high malaria transmission, most symptomatic malaria and nearly all severe malaria and malaria deaths are in young children. This is the pattern in much of the malaria-endemic areas of West Africa, which bear the brunt of global malaria morbidity and mortality. Across the Sahel transmission of malaria is intense during the 3–4 rainy season months, then it declines to almost zero in the longer dry season. In these areas symptomatic malaria is a rainy season disease of young children. Older children become progressively less likely to be ill when infected, and adults are largely asymptomatic, yet at any time a high proportion of the community harbors malaria parasites in their blood. How important is this asymptomatic parasite carriage in maintaining immunity and thus protection against malaria illness? In this issue of *Clinical Infectious Diseases*, Portugal et al. address this question in a detailed longditudinal cohort study conducted in Kalifabougou, a rural village in Mali of ~5000 inhabitants with intense rainy season transmission of malaria typical of much of the Sahel region [1]. In the dry season between December and July there was almost no malaria transmission and therefore no symptomatic malaria, yet 46% of villagers aged between 6 months and 25 years had *P. falciparum* parasitemia detected by a polymerase chain reaction (PCR) assay, which has a limit of detection of 500–1000 parasites/mL. Genotyping in this and other studies strongly suggests that these parasites were carried in the blood throughout the dry season. Several studies have shown that detectable parasitemia before the rainy season is associated with a lower risk of symptomatic malaria during it. If these chronic infections were important in maintaining protective immunity, then treating them might predispose to more severe infections in the next rainy season. This prospective study shows clearly that this is not the case. Treatment of these asymptomatic infections with artemether-lumefantrine was not associated with an increased risk of symptomatic malaria in the following two rainy seasons. Furthermore, the decline in antibody profiles against 862 different *P. falciparum* parasite proteins was similar in infected and uninfected individuals. There seems no downside, at least in the near term, to reducing asymptomatic malaria.

So should we be treating everyone with antimalarial drugs during the dry season in these areas of seasonal high transmission? In addition to the deployment of insecticide treated bed nets and prompt diagnosis and treatment of symptomatic malaria, the primary strategy currently recommended to reduce morbidity and mortality from falciparum malaria across the Sahel is seasonal malaria chemoprevention (SMC). This involves giving monthly treatment doses of amodiaquine and sulfadoxine-pyrimethamine to all children under 5 years of age during the 3–4 month rainy season. SMC has been shown to be 75% protective against uncomplicated and severe malaria. It is cost-effective and it is safe and can be administered by community-health workers. An estimated 25 million west African children aged between 3 months and 5 years could benefit from SMC every year. The role of dry season mass treatments is unclear. The weight of current opinion favors use of this approach only for elimination (ie, in areas where malaria transmission is generally low) or for epidemic containment. In low-transmission settings asymptomatic carriage rates can still be high, and mass treatment can be used to eliminate malaria rapidly. However, one of the arguments against this approach is that mass treatments would “interfere with immunity and predispose to severe malaria;” a similar concern to that in high transmission settings. But in this very different context the objective is to eliminate malaria, so it is inevitable that any protective immunity will eventually be lost if the effort is successful—emphasizing the critical importance of sustaining elimination effectively once it has been achieved.

## Note

***Potential conflicts of interest.*** Author certifies no potential conflicts of interest. The author has submitted the ICMJE Form for Disclosure of Potential Conflicts of Interest. Conflicts that the editors consider relevant to the content of the manuscript have been disclosed.
